# Supplementary material for: Engineering functional ventral midbrain dopaminergic neurons in human organoids through WNT modulation and bioreactor culture
Source: Mol Psychiatry. 2026 Jun 17;31(10):5900–16. doi: 10.1038/s41380-026-03667-4 (PMC13569470; doi:10.1038/s41380-026-03667-4)
Supplement: Supplementary file 1 [file 41380_2026_3667_MOESM10_ESM.pdf]

Supplementary Table 2

Primary and secondary antibodies

Primary antibodies	Species	Manufacturer + Identifier	Dilution	RRID
Anti-MAP2 antibody	chicken	BioLegend Cat # 822501	1:2500	RRID:AB_2564858
Anti-Cleaved Caspase-3	rabbit	Cell Signaling Cat # 9664	1:500	RRID:AB_2070042
Tyrosine Hydroxylase	rabbit	Pel-Freez Cat # P40101-150	1:500	RRID:AB_2617184
Tyrosine Hydroxylase	sheep	Pel-Freez Cat # P60101-150	1:450	RRID:AB_3677426
Alpha Synuclein	rabbit	Abcam Cat # ab138501	1:2500	RRID:AB_2537217
GIRK2 (KCNJ6)	rabbit	Millipore Cat # AB5200	1:250	RRID:AB_91747
VMAT-2	goat	Santa Cruz Cat # sc-7721	1:500	RRID:AB_10611330
ALDH1A1	goat	R&D Systems Cat # AF5869	1:100	RRID:AB_2044597
GFAP	rabbit	DAKO Cat # Z0334	1:1000	RRID:AB_10013382
SOX2	mouse	Abcam Cat # ab75485	1:250	RRID:AB_1278243
OTX2	rabbit	Proteintech Cat # 13497-1-AP	1:250	RRID:AB_2157176
B-Tubulin IIII (Tuj1)	mouse	Biolegend Cat # 801201	1:1000	RRID:AB_2313773
HNF-3 beta /FoxA2	goat	R&D Systems Cat # AF2400	1:100	RRID:AB_2294104
EN1	rabbit	Millipore Cat # AB5732	1:200	RRID:AB_2098599
LMX1A	rabbit	Abcam Cat # ab31006	1:250	RRID:AB_881120
Calbindin	mouse	Swant Cat # 300	1:450	RRID:AB_10000347
DAT	rat	Santa Cruz Cat # sc32259	1:200	RRID:AB_627402
DDC	rabbit	Invitrogen Cat # PA5-25450	1:250	RRID:AB_2542950
Synaptophysin	mouse	Proteintech Cat # 67864-1	1:500	RRID:AB_2918622
PSD-95	rabbit	Proteinttech Cat # 20665-1	1:250	RRID AB_2687961

Secondary antibodies	Species	Manufacturer + Identifier	Dilution
Anti-Rabbit Alexa Fluor 568	Goat	Invitrogen Cat # A-11011	1:1000
Anti-Chicken Alexa Fluor 647	Goat	Invitrogen Cat # A-48260	1:1000
Anti-Mouse Alexa Fluor 488	Goat	Invitrogen Cat # A-28175	1:1000
Anti-Mouse Alexa Fluor 647	Goat	Invitrogen Cat # A-21235	1:1000
Anti-Rabbit Alexa Fluor 488	Goat	Invitrogen Cat # A-11008	1:1000
Anti-Mouse Alexa Fluor 488	Donkey	Invitrogen Cat # R-37114	1:1000
Anti-Rabbit Alexa Fluor 568	Donkey	Invitrogen Cat # A-10042	1:1000
Anti-Sheep Alexa Fluor 488	Donkey	Invitrogen Cat # A-11015	1:1000
Anti-Sheep Alexa Fluor 647	Donkey	Invitrogen Cat # A-21448	1:1000
Anti-Mouse Alexa Fluor 647	Donkey	Invitrogen Cat # A-31571	1:1000
Anti-Goat Alexa Fluor 488	Donkey	Invitrogen Cat # A-11055	1:1000
Anti-Rabbit Alexa Fluor 488	Donkey	Invitrogen Cat # A-21206	1:1000
