# Supplementary material for: Engineering functional ventral midbrain dopaminergic neurons in human organoids through WNT modulation and bioreactor culture
Source: Mol Psychiatry. 2026 Jun 17;31(10):5900–16. doi: 10.1038/s41380-026-03667-4 (PMC13569470; doi:10.1038/s41380-026-03667-4)
Supplement: Supplementary file 5 [file 41380_2026_3667_MOESM5_ESM.pdf]

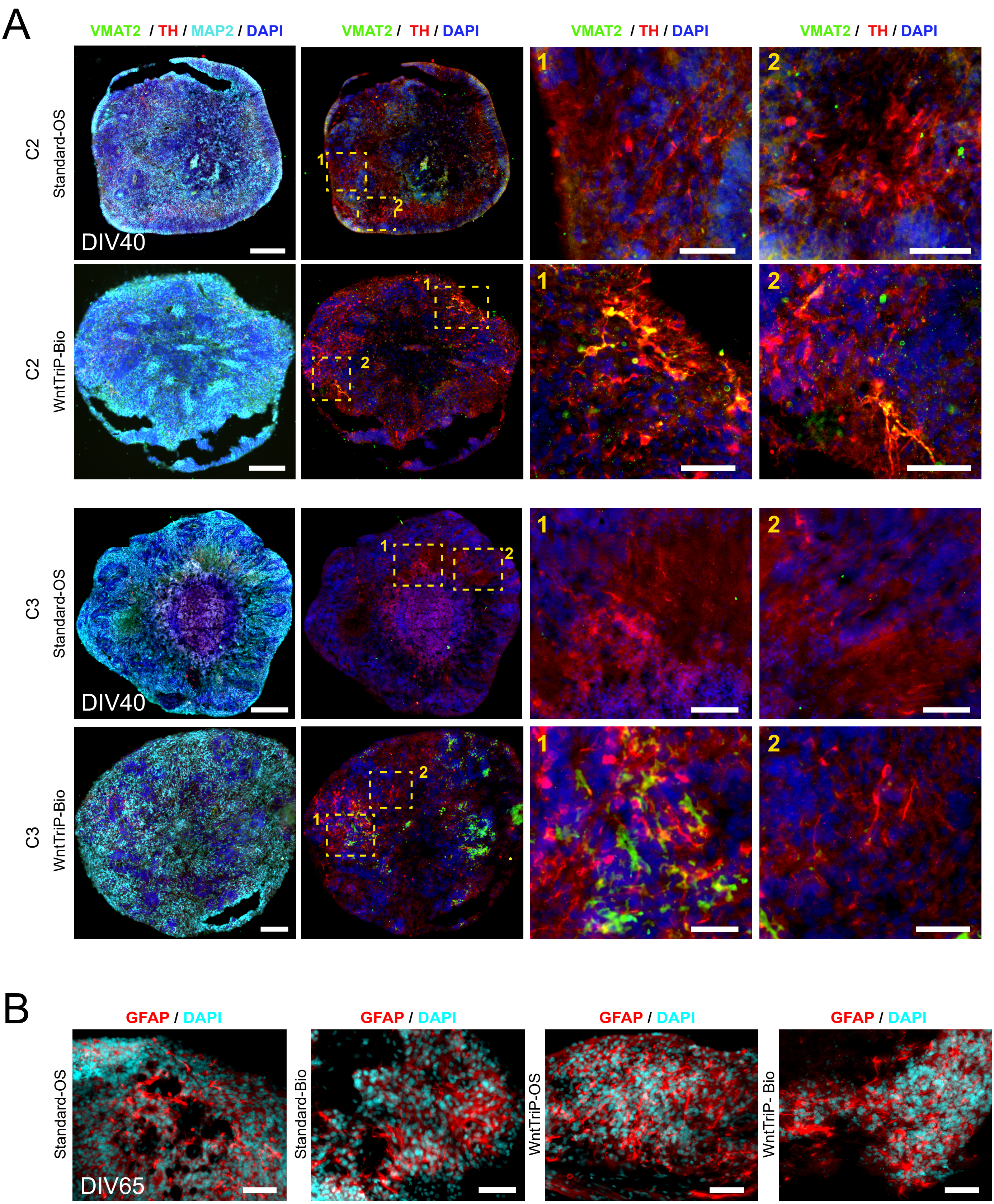

Supplementary Figure 4
