# Supplementary material for: Engineering functional ventral midbrain dopaminergic neurons in human organoids through WNT modulation and bioreactor culture
Source: Mol Psychiatry. 2026 Jun 17;31(10):5900–16. doi: 10.1038/s41380-026-03667-4 (PMC13569470; doi:10.1038/s41380-026-03667-4)
Supplement: Supplementary file 8 [file 41380_2026_3667_MOESM9_ESM.pdf]

Supplementary Table 1

Materials and reagents

Materials and reagents	Manufacturer	Catalog Number	Location
mTeSR1 medium	StemCell Technologies	Cat # 85850	Vancouver, Canada
Matrigel	Corning	Cat # 354230	New York, USA
Penicillin-streptomycin solution	ThermoFisher	Cat # 15140122	Waltham, MA, USA
ReLeSR dissociation agent	StemCell Technologies	Cat # 100-0483	Vancouver, Canada
Venor®GeM Classic Mycoplasma detection kit	Minerva-Biolabs	Cat # 11-1025G	Berlin, Germany
Neurobasal medium	ThermoFisher	Cat # 21103049	Waltham, MA, USA
N-2 supplement	ThermoFisher	Cat # 17502048	Waltham, MA, USA
B27 supplement without vitamin A	ThermoFisher	Cat # 12587010	Waltham, MA, USA
GlutaMax	ThermoFisher	Cat # 35050061	Waltham, MA, USA
Non-Essential Amino Acids (NEAA)	ThermoFisher	Cat # 11140050	Waltham, MA, USA
SHH-C25II	R&D Systems	Cat # 1845-SH-025	Minneapolis, MN, USA
FGF8	R&D Systems	Cat # 423-F8-025	Minneapolis, MN, USA
BDNF	PeproTech	Cat # 450-02	Rocky Hill, NJ, USA
GDNF	PeproTech	Cat # 450-10	Rocky Hill, NJ, USA
Low-attachment U-bottom 96-well plates	Corning	Cat # 7007	New York, USA
48-well cell culture plate	Corning	Cat # 353078	New York, USA

Materials and reagents	Manufacturer	Catalog Number	Location
Customized SpinΩ Bioreactor – 12-well	3Dnamics	Cat # 3DNA01	Baltimore, MD, USA
HeracellTM 240i CO2 Incubator	ThermoFisher	Cat # 16426639	Waltham, MA, USA
Superfrost PLUS tissue adhesion glass slides	Epredia	Cat # J1800AMNZ	Kalamazoo, MI, USA
Leica CM3050S cryostat	Leica Biosystems	N/A	Germany
Leica TCS SP8 confocal microscope	Leica Biosystems	N/A	Germany
Zeiss Spinning Disk confocal microscope	ZEISS	N/A	Germany
BioCAM DupleX system with Accura-3D HD-MEA chips	3Brain	N/A	Pfaeffikon, Switzerland
DAKO mounting medium	Agilent DAKO	Cat # S302380-2	Glostrup Municipality, Denmark
CHIR99021	Tocris Bioscience	Cat # 4423	Bristol, UK
SB431542	Tocris Bioscience	Cat # 1614	Bristol, UK
LDN	Tocris Bioscience	Cat # 6053	Bristol, UK
IWP-2	StemCell Technologies	Cat # 72124	Vancouver, Canada
Purmorphamine	Tocris Bioscience	Cat # 4551	Bristol, UK
Rock inhibitor (Y-27632)	Tocris Bioscience	Cat # 1254	Bristol, UK
β-mercaptoethanol	ThermoFisher	Cat # 21985023	Waltham, MA, USA
Heparin	Sigma-Aldrich	Cat # H3149	St. Louis, MO, USA
Insulin	Sigma-Aldrich	Cat # I1882	St. Louis, MO, USA

Materials and reagents	Manufacturer	Catalog Number	Location
Laminin	Sigma-Aldrich	Cat # L2020	St. Louis, MO, USA
Ascorbic acid	Sigma-Aldrich	Cat # A4544	St. Louis, MO, USA
dbcAMP	Sigma-Aldrich	Cat # D0627	St. Louis, MO, USA
Accutase	StemCell Technologies	Cat # 07920	Vancouver, Canada
Papain Dissociation System kit	Worthington Biochemical	Cat # LK003150	Lakewood, NJ, USA
10X Chromium Next GEM Single Cell 3’ Reagent Kit v3.1 Dual Index	10xGenomics	Cat. 1000123	Pleasanton, CA, USA
4-Aminopyridine	Sigma-Aldrich	Cat. 275875	St. Louis, MO, USA
DeadEnd™ Fluorometric TUNEL System	Promega	Cat. G3250	Madison, WI, USA
PE/Atto 594 Conjugation Kit - Lightning-Link®	Abcam	Cat # ab269900	Cambridge, UK
Proteinase K solution	New England Biolabs	Cat # P8107S	MA, USA
